# Native and Complexed IGF-1: Biodistribution and Pharmacokinetics in Infantile Neuronal Ceroid Lipofuscinosis

**DOI:** 10.1155/2012/626417

**Published:** 2012-06-15

**Authors:** Tuulia Huhtala, Jussi Rytkönen, Anu Jalanko, Martti Kaasalainen, Jarno Salonen, Raili Riikonen, Ale Närvänen

**Affiliations:** ^1^A.I. Virtanen Institute, University of Eastern Finland, 70211 Kuopio, Finland; ^2^Biocenter Kuopio, University of Eastern Finland, 70211 Kuopio, Finland; ^3^School of Pharmacy, University of Eastern Finland, 70211 Kuopio, Finland; ^4^National Institute for Health and Welfare, Public Health Genomics Unit, Biomedicum Helsinki, Haartmaninkatu 8, 00290 Helsinki, Finland; ^5^Department of Physics and Astronomy, University of Turku, 20014 Turku, Finland; ^6^Department of Pediatrics, Kuopio University Hospital, 70211 Kuopio, Finland

## Abstract

Infantile neuronal ceroid lipofuscinosis (INCL) is a severe neurodegenerative disorder of childhood characterized by selective death of cortical neurons. Insulin-like growth factor 1 (IGF-1) is important in embryonic development and is considered as a potential therapeutic agent for several disorders of peripheral and central nervous systems. In circulation IGF-1 is mainly bound to its carrier protein IGFBP-3. As a therapeutic agent IGF-1 has shown to be more active as free than complexed form. However, this may cause side effects during the prolonged treatment. In addition to IGFBP-3 the bioavailability of IGF-1 can be modulated by using mesoporous silicon nanoparticles (NPs) which are optimal carriers for sustained release of unstable peptide hormones like IGF-1. In this study we compared biodistribution, pharmacokinetics, and bioavailability of radiolabeled free IGF-1, IGF-1/IGFBP-3, and IGF-1/NP complexes in a *Cln1*-/- knockout mouse model. IGF-1/NP was mainly accumulated in liver and spleen in all studied time points, whereas minor and more constant amounts were measured in other organs compared to free IGF-1 or IGF-1/IGFBP-3. Also concentration of IGF-1/NP in blood was relatively high and stable during studied time points suggesting continuous release of IGF-1 from the particles.

## 1. Introduction 

Infantile neuronal ceroid lipofuscinosis (INCL) is a severe neurodegenerative disorder of childhood characterized by selective death of cortical neurons [1]. Treatment is focused mainly to relieve the symptoms, such as sleep difficulties and epilepsy, but the average lifespan of an INCL child is still only 10 years. INCL is caused by recessive mutations in the *CLN1* gene encoding palmitoyl-protein thioesterase (PPT1) [2]. Normal PPT1 activity is essential for the development and survival of cortical and cerebellar neurons in human and mouse [3–5]. IGF-1 concentration in cerebrospinal fluid is lower in patients with INCL [3] suggesting that decreased levels of IGF-1 in brain may accelerate neurodegenerative disorders. To consistently study pathogenesis and treatment of INCL and other types of neuronal ceroid lipofuscinoses (NCLs), different mouse models have been established (CLN1, CLN2, CLN3, CLN5) and also naturally occurring NCL mouse models exist (CLN8/mnd; CLN6/nclf) [6]. The *Cln1-/-* knockout mouse model is analogous to INCL in humans, with severe phenotype, and the overall neurologic features are highly similar to the clinical symptoms of INCL [4]. Furthermore, the analyses of two different *Cln1-/-* mouse models have revealed new pathological characteristics for INCL, including early thalamocortical neuron loss accompanied by astrocytosis, defects in axonal growth, cholesterol biosynthesis, and calcium metabolism [7–10].

Insulin-like growth factors, IGF-1 and IGF-2, are members of the insulin gene family and they play an important role in physiological development of humans and animals. IGF-1 and -2 stimulate cell proliferation and differentiation during embryonic and postnatal development. IGF-1 is the main trophic factor in the central nervous system (CNS) during early brain development [11, 12] and its relevance is greater to IGF-2. IGF-1 stimulates DNA synthesis, cell proliferation, neurite outgrowth, axonal growth, and myelination and enhances secretion of various neurotransmitters. IGF-1 signals many neurodegenerative diseases [13, 14] and lack of IGF-1 in the brain leads to apoptosis. IGF-1 knockout mice show microcephaly and demyelination of the whole brain [15] and overstimulation of IGF-1 leads to macrocephaly [16]. In animal experiments neurotrophins have shown to have therapeutic effects on motor neuron disorder [17], chemotherapy-induced peripheral neuropathy [18], myelination and brain growth [19], asphyxia [20], cerebellar ataxia [21], retinopathy of prematurity [22], cognitive impairment [23], and experimental autoimmune encephalitis [24, 25]. It has been shown that only one-week treatment with IGF-1 partially restored interneuronal number and reduced hypertrophy in the *mnd/mnd* mouse model of neuronal ceroid lipofuscinosis (CLN8) [26, 27].

IGF-1 is a polypeptide containing 70 amino acids, with a molecular weight of only 7.6 kDa. It is associated with one of the six known high-affinity binding proteins (IGFBP-1 to 6). They have a central role in transporting IGFs in the bloodstream and cerebrospinal fluid and across the capillary barrier to the target cells [28] associating directly with cell membranes [29]. IGFBPs increase the half-time and stabilizing the IGF stimulation [30]. IGFBP-3 (MW 150 kDa) is the predominant IGFBP in serum. Most of the circulating IGF-1 and IGF-2 form a ternary complex with IGFBP-3 and acid labile subunit (ALS) [31].

Mesoporous silicon (PSi) micro- and nanoparticles are promising drug carriers for the targeted therapy for example, due to their high payload of therapeutic agents and biocompatibility [32, 33]. Depending on the size and the surface chemistry of the pores increased or sustained release of the loaded therapeutic agents can be adjusted [33, 34]. Porous silicon has already been utilized in the delivery of biologically unstable molecules such as peptides [35].

We have studied the biodistribution of I-125 labeled free IGF-1, IGF-1/IGFBP-3, and IGF-1/NP complexes in *Cln1-/-* mouse model which genotypically and phenotypically represents INCL. Accumulation of free or complexed IGF-1 in selected organs was measured at three time points. The aim of this study was to compare accumulation and pharmacokinetics of free and complexed IGF-1 to the brain in order to evaluate the therapeutic potential for INCL. 

## 2. Materials and Methods 

### 2.1. Radiolabeling

IGF-1 and IGFPB-3 were offered by Insmed Incorporation (Richmond, VA, USA). IGF-1 and IGFBP-3 were radiolabeled with ^125^I with Iodo-Gen method. Briefly, precoated iodination tubes (Pierce) were rinsed with 1 mL of phosphate-buffered saline, pH 7.4, (PBS), and ^125^I (22 MBq, Map Medicals, Finland) was incubated at room temperature for 10 minutes. After incubation IGF-1 (200 *μ*g) or IGFBP-3 (200 *μ*g) was added and the reaction mixture was further incubated for 15 minutes at RT. The solution was purified using HiTrap Sephadex column (GE Healthcare) using PBS as a mobile phase at flow rate 1 mL/min. Labeling efficiency was 29–43% with specific activity of 0.22 MBq/nmol and 0.37 MBq/nmol for the IGF-1 and 11–17% for the IGFBP-3 with specific activity of 0.29 MBq/nmol and 0.33 MBq/nmol, respectively. 

### 2.2. Nanoparticles

Thermally hydrocarbonized mesoporous silicon nanoparticles (THCPSi) were prepared as described earlier [36]. Nanoparticles (800 *μ*g) were mixed with radiolabelled IGF-1 (200 *μ*g) in 2 mL of 10 mM HEPES pH 7.4. The suspension was mixed at RT for two hours sonicating every 30 minutes. 94% of IGF-1 was incorporated in the particles and the loading degree was 23.5% (w/w). The *in vitro *release was studied using fresh mouse plasma diluted 1 : 2 in PBS. Nanoparticles were mixed with diluted plasma and incubated at +37°C. A sample of the particles was centrifuged immediately and at 20, 60, 120, and 240 minutes time points (*n* = 3/time point). Radioactivity of the supernatant was measured by Gamma Counter (1277 Gammamaster automatic Gamma Counter, LKB Wallac, Finland).

### 2.3. Animals

A homozygous knockout mouse model *Cln1-/-,* showing overall neurologic features highly similar to the clinical symptoms of INCL, was used in this study [4]. The *Cln1-/-* mice were backcrossed to *C57BL/6* for more than 10 generations, and the congeneity was confirmed with the Mouse Medium Density SNP Panel (Illumina). The genotypes of the mice were determined by PCR of DNA from tail biopsies. Total of 36 nine-week-old (*n* = 3/group) female mice were used for the biodistribution studies. The mice received chow and water *ad libitum*. All animal procedures were performed according to protocols approved by the ethical boards for animal experimentation of the National Public Health Institute and University of Helsinki, as well as National Animal Experiment Board of Regional State Administrative Agencies of Southern Finland (Agreement number 09-06737), and all experiments were done in accordance with good practice of handling laboratory animals and genetically modified organisms.

### 2.4. Biodistribution Studies in *Cln1-/-* Mice

The biodistribution of radioiodinated IGF-1 was studied using free IGF-1, IGF-1 complexed with unlabeled IGFBP-3 (IGF-1/IGFPB-3), or with nanoparticles (IGF-1/NP). In addition, the biodistribution of plain IGFBP-3 was studied. IGF-1 was complexed with IGFBP-3 using 2 : 1 molar ratio in PBS. The final protein concentration for the three protein preparations was 0.1 mg/mL.

Animals were anesthetized by 1.5–2% isoflurane in N_2_/O_2_ with ratios 70 : 30, respectively. Labeled IGF-1, IGF-1/IGFPB-3, IGF-1/NP, or IGFBP-3 was injected i.v. via tail vein using 10 *μ*g of IGF-1; 0.2–0.6 MBq/animal. Also 1 mL of 5% glucose was administrated i.p. to prevent hypoglycemia. Animals were sacrificed at 20, 120, or 240 min after injection. Tissue samples were collected in tared tubes and radioactivity was measured using automated gamma counter (Clinigamma, Wallac, Finland). Corrections were made for background radiation and physical decay during counting. The activity in all organs and tissue samples was expressed as percentage of injected dose per gram (%ID/g). All data were expressed as mean ± standard error of the mean (S.E.M).

## 3. Results 

Biodistribution of radiolabeled native IGF-1 was compared to IGF-1/IGFBP-3 and IGF-1/NP complexes and free IGFBP-3 in *Cln1-/-* mouse model. The animals were sacrificed 20, 120, or 240 min after injection and radioactivity of selected organs was measured using a Gamma Counter (Table 1).

### 3.1. The In Vitro Release of IGF-1 from Nanoparticles

**Figure 1:**
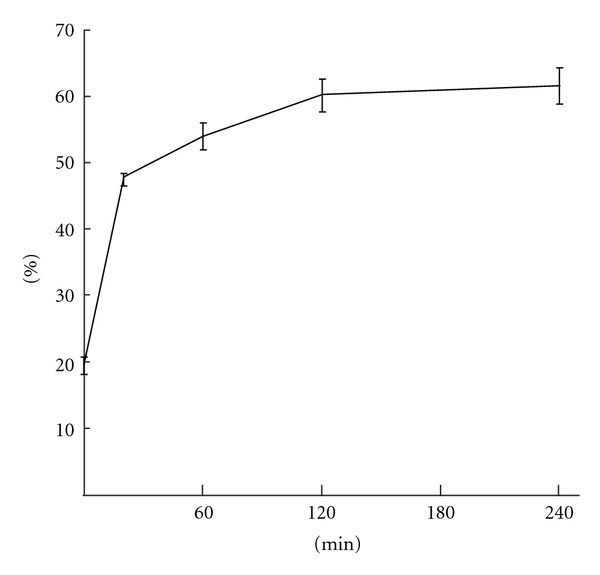
*In vitro* release of 125-I labelled IGF-1 from nanoparticles. 20% of the IGF-1 was burst immediately after mixing with mouse plasma and 50% was released after 20 minutes incubation at 37°C. Only 15% was released between 20 and 240 min time points.

The release kinetics of IGF-1 from THCPSi nanoparticles was studied *in vitro* in mouse plasma at +37°C. As shown in Figure 1 there was a burst immediately after mixing with the plasma releasing 20% of the incorporated IGF-1. After 20 minutes half of the IGF-1 was released and detachment rate was further decreased, 60% of IGF-1 was uncoupled at 240 min time point.

### 3.2. Clearance and Bioavailability of the Native and Complexed IGF-1

**Figure 2:**
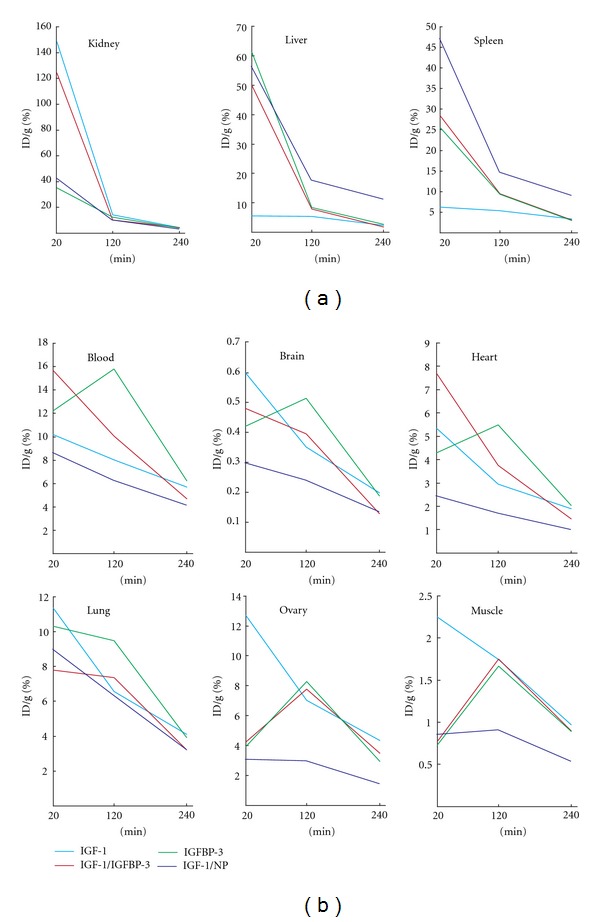
(a) Clearance of unbound and complexed IGF-1 *in vivo*. (b) Pharmacokinetics of free and complexed IGF-1 in selected organs.

As expected, most of the unbound IGF-1 was cleared through the kidneys within 120 minutes (Figure 2(a)). The clearance of IGF-1/IGFBP-3 through the kidneys was also high indicating fast dissociation since the labeled IGFBP3 was mainly excreted through the hepatic route. However, substantial part (50.1% ID/g) of IGF-1/IGFBP-3 was also eliminated through the liver after 20 min. Excretion of IGF-1/NP via the kidneys was significantly inferior to unbound IGF-1 or IGF-1/IGFBP-3 (42.3% ID/g; 148.3 and 124.2% ID/g) indicating sustained release of IGF-1 from the nanoparticles. 

At 20 min time point accumulations of IGF-1/IGFBP-3 and IGF-1/NP in the liver were nearly equal (50.1% ID/g; 56.5% ID/g). Interestingly, concentration of IGF-1/IGFBP-3 in liver decreased to the level of IGF-1 between 20 and 120 min whereas the accumulation of IGF-1/NP was 2 times higher after 120 min and 5 times higher after 240 min than corresponding IGF-1/IGFBP-3 or protein alone (Figure 2(a)). Protein complexed IGF-1 was removed by active excretion whereas IFG-1/NP complex is concentrated into the liver and rather dissociated to the circulation than excreted immediately. 

The level of IGF-1/IGFBP-3 in blood decreased faster in both time frames 20–120 min (36%) and 120–240 min (53%) than IGF-1/NP (Table 1). Concentration of IGF-1/NP in blood stayed steadier between both time frames (20–120 min, 27%; 120–240 min, 34%) like unbound IGF-1 (Figure 1(b)). This suggests that IGF-1/NP is concentrated in liver and rather dissociated to circulation enabling longer bioavailability to other tissues than excreted immediately. 

### 3.3. Brain

IGF-1 accumulated relatively low levels in the brain with or without complex in all studied time points (Figure 2(b)). The highest accumulation of IGF-1 was achieved at 20 min time point (0.60% ID/g) which was 25% more than IGF-1/IGFBP-3 (0.48% ID/g) and twice as much as IGF-1/NP (0.30% ID/g). However, accumulation of IGF-1 drops dramatically between 20–120 min (42%), whereas levels of IGF-1/IFGBP-3 or IGF-1/NP decreased only 17% and 18%, respectively, indicating more constant delivery of the IGF-1 in the brain. This data shows that the complexed forms of IGF-1 does not enhance delivery of IGF-1 in the brain but gives more stable concentrations. After 240 min IGF-1 accumulation in the brain was the same with or without complex.

### 3.4. Pharmacokinetics in Other Selected Tissues

Although the concentration of IGF-1/NP in blood was similar to the freely injected IGF-1 at all studied time points the accumulation of IGF-1/NP was lower than free IGF-1 and IGF-1/IGFBP-3 in most of the studied organs, which may result in milder side effects. Pharmacokinetics of free IGF-1 and IGF-1/NP showed linear clearance during the first 240 min in all studied organs (Figure 2(b)), whereas the kinetics of IGF-1/IGFBP-3 depended on the organ. In the ovaries and muscle the maximal activity was found at 120 minutes time point and at the remaining organs the highest accumulation was reached in 20 min. Concentration of IGFBP-3 was highest at 120 min in all studied organs.

## 4. Discussion 

The maturation of the BBB is species dependent. In some animals, the BBB matures during the earliest stages of gestation, while in others just before birth, and yet others not until after birth. In human infants, BBB maturation is complete approximately 6–12 months after birth [37, 38]. It is not known whether the BBB was already completed in the mice of the present study (at the age of 8–9 weeks of age). However, to be effective therapy, firstly, the drug should be given at an early age to prevent the damage and when BBB is still open. In animals, IGF-1 treatment strongly affects CNS myelination at an early age, but is ineffective later on, suggesting that there is critical period for CNS myelination [39]. Secondly, it must be noted that neurodegeneration and clinical course (maturation), are much faster in mice than in humans, for example, loss of vision occurs in mutant mouse at the age of 8 weeks and in humans at 8–18 months, respectively. 

There is no direct evidence about the therapeutic effect of IGF-1 in mice. However, the effect of IGF-1 has been tested in several human disorders. Positive effects in adults have already been shown in body and brain growth [40–42], insulin resistance [43], and head injury [44]. In children it was used in growth hormone insensitivity syndrome (Laron syndrome). In adolescents with type 1 diabetes it has been used to promote insulin sensitivity. Recently, a study of IGF-1 as therapeutic agent in Rett syndrome have been initiated in Children's hospital, Boston (Khwaja, Clinical Trials.Gov ID NCT01253317). Also growth hormone treatment which has its main effect through IGF-1 has shown to be favorable to the motor and cognitive effects in CP [45]. 

The activity of free IGF-1 has been reported to be more active than IGF-1/IGFBP-3 [46]. However, undesired acute adverse reactions and the absence of suitable IGF-1 preparations for treatment have become major concerns among pediatric endocrinologists worldwide. The main problems in IGF-1 treatment include BBB permeability, side-effects like hypoglycemia, and short intervals in administration of the drug. IGF-1 complexed with binding protein 3 (Somatokine R) used in this study was developed to prolong the half-life and reducing side effects (including hypoglycemia). Recombinant hormone seems to be safe even in prolonged therapy for growth hormone insensitivity syndrome or for short children [47]. 

Our aim was to compare the biodistribution of free IGF-1, IGF-1/IGFBP-3, and IGF-1/NP complex in selected organs over the time in *Cln1-/-* mouse model if IGF-1 could be used as a potential drug to treat INCL. It has been suspected that IGF-1/IGFBP-3 is too large to cross the BBB, but since IGF-1 is shed, it may eventually cross the BBB by specific transport systems [48, 49]. In *Cln1-/-* mice there is inflammation, and prominent alterations involved in the immune response [4, 9]. Consequently, in the INCL patients, there might be inflammatory changes that could open BBB and the IGFs could cross it better than expected in healthy people.

Earlier studies in mice have shown that IGF-1 injected as such is bound to the plasma proteins immediately after the injection forming different size of complexes and is cleared via the kidneys [50, 51]. In our biodistribution and pharmacokinetic studies iodinated IGF-1 was complexed to IGFBP-3 or nanoparticles* in vitro* before the injection. IGF-1 accumulated strongly in the liver only as a protein or nanoparticle complex. However, the protein complex was mainly excreted after two hours in contrast to IGF-1/NP which accumulation in liver was over 17% ID/g at the same time point. It has been shown that intravenously administered mesoporous silicon microparticles loaded with siRNA encapsulated into nanoliposomes accumulate into the liver and spleen, but remain in the sinusoidal space, enabling sustained release of siRNA-loaded nanoliposomes [52].

In our other studies we have analyzed behavior of I-125 labeled thermally hydrocarbonized mesoporous silicon nanoparticles in liver using combined data of autoradiography and electron microscopy [53]. Similar nanoparticles as used in this study were seen in hepatic veins and sinusoids but not internalized into macrophages or hepatocytes. In addition, Bimbo et al. reported that THCPSi nanoparticles are not phagocytes in extent by CaCo-2 or RAW 264.7 macrophages *in vitro*. Instead they showed a strong cellular association as majority of the nanoparticles remained attached to cell membranes [54]. We suggest that IGF-1/NP is intact in the liver and IGF-1 may be released during the 240 min studied, whereas IGF-1/IGFBP-3 is cleared trough hepatic system. This can be seen as steadier IGF-1 release in blood compared IGF-1/NP to IGF-1/IGFBP-3 and is also in accordance with our *in vitro* results. More stable release of IGF-1 conjugated to NPs can be achieved as compared to protein complexes.

The doses used in our study have been the same as in experimental autoimmune encephalomyelitis mice where positive effects on inflammatory, demyelinating, and demyelinated lesions have been seen when using IGF-1 [55]. Relatively low levels of IGF-1 with or without IGFBP-3 or nanoparticles accumulated to the brains in all studied time points. The amount crossing the BBB might, however, be sufficient to affect the physiological functions and modulate neuroendocrine and behavioural responses. The sustained release to blood and low tissue concentrations of IFG-1 delivered with nanoparticles may decrease the side effects like hypoglycemia without losing the therapeutic effect. Low blood and tissue concentrations together with constant and sustained release may be beneficial for the continuous IGF-1 therapy for INCL.

In summary, we have studied the biodistribution and pharmacokinetics of human IGF-1 administrated free or complexed to its natural binding protein IGFBP-3 or nanoparticles in infantile neuronal ceroid lipofuscinosis (INCL) mouse model. IGF-1 conjugated to nanoparticles accumulated and also remained in liver probably in the hepatic veins and sinusoids at high concentration in contrast to IGF-1/IGFPB-3 complex which dissociated and was actively excreted via kidneys and liver during studied time points. Since IGF-1/NP level also in blood decreased moderately compared to IGF-1/IGFBP-3 this data demonstrates steadier release of IGF-1 in to the circulation and longer bioavailability of IGF-1. Also IGF-1/NP concentration in all other studied tissues except spleen was lower than with protein complex or unbound IGF-1 which may result in milder side effects and is beneficial for the long-term hormone therapy. 

## Figures and Tables

**Table 1:** Biodistribution of unbound IGF-1, IGF-1/IGFBP-3, IGFBP-3, and IGF1/NP complex 20 min, 120 min, and 240 min post-i.v. injection in *CLN1-/-* mice. The activity in all organs and tissue samples is expressed as percentage of injected dose/tissue sample weight (%ID/g). All data are expressed as mean ± standard error of the mean (S.E.M).

Organ	IGF-1 complex			
	IGF-1	IGF-1/IGFBP-3	IGFBP-3	IGF-1/NP
20 min				
Blood	10.21 ± 0.92	15.69 ± 0.90	12.24 ± 0.49	8.66 ± 0.04
Heart	5.35 ± 0.49	7.70 ± 0.23	4.29 ± 0.55	2.45 ± 0.06
Lung	11.37 ± 1.00	7.81 ± 0.24	10.30 ± 1.95	9.00 ± 0.62
Kidney	148.3 ± 34.48	124.2 ± 9.90	34.98 ± 8.90	42.96 ± 3.83
Liver	5.52 ± 0.50	50.06 ± 2.21	61.19 ± 16.80	56.45 ± 8.44
Spleen	6.22 ± 0.39	28.37 ± 1.22	25.50 ± 13.71	47.15 ± 4.11
Ovary	12.71 ± 1.46	4.22 ± 0.57	3.92 ± 0.52	3.09 ± 0.45
Muscle	2.25 ± 0.24	0.77 ± 0.05	0.73 ± 0.06	0.85 ± 0.00
Brain	0.60 ± 0.04	0.48 ± 0.04	0.42 ± 0.00	0.30 ± 0.00

120 min				
Blood	8.01 ± 0.92	10.05 ± 2.66	15.82 ± 2.01	6.30 ± 0.21
Heart	2.95 ± 0.45	3.76 ± 1.22	5.50 ± 0.50	1.71 ± 0.08
Lung	6.57 ± 0.97	7.35 ± 2.42	9.49 ± 1.06	6.37 ± 0.57
Kidney	14.32 ± 2.23	10.31 ± 4.21	12.40 ± 1.19	10.27 ± 1.97
Liver	5.31 ± 0.74	7.95 ± 4.78	8.45 ± 1.28	17.75 ± 0.83
Spleen	5.30 ± 0.56	9.45 ± 3.28	9.40 ± 3.28	14.71 ± 2.49
Ovary	7.04 ± 1.66	7.78 ± 2.14	8.27 ± 0.55	2.99 ± 0.11
Muscle	1.74 ± 0.43	1.75 ± 0.48	1.67 ± 0.07	0.91 ± 0.03
Brain	0.35 ± 0.08	0.40 ± 0.16	0.51 ± 0.04	0.24 ± 0.01

240 min				
Blood	5.71 ± 0.41	4.68 ± 0.17	6.24 ± 0.43	4.15 ± 0.39
Heart	1.89 ± 0.15	1.46 ± 0.08	2.03 ± 0.13	1.00 ± 0.12
Lung	4.10 ± 0.46	3.23 ± 0.03	3.93 ± 0.46	3.25 ± 0.49
Kidney	4.20 ± 0.25	4.36 ± 1.14	3.87 ± 0.31	3.08 ± 0.42
Liver	2.29 ± 0.19	1.79 ± 0.17	2.74 ± 0.10	11.29 ± 0.63
Spleen	3.41 ± 0.32	3.16 ± 0.38	2.93 ± 2.93	9.05 ± 1.01
Ovary	4.33 ± 0.43	3.49 ± 0.16	2.94 ± 0.43	1.47 ± 0.03
Muscle	0.97 ± 0.06	0.90 ± 0.14	0.89 ± 0.19	0.54 ± 0.03
Brain	0.20 ± 0.01	0.13 ± 0.01	0.19 ± 0.01	0.14 ± 0.01
